# Microfluidic flowmeter based on a liquid crystal-filled nested capillary

**DOI:** 10.1038/s44172-024-00202-7

**Published:** 2024-03-21

**Authors:** Zhe Wang, Arun Kumar Mallik, Fangfang Wei, Zhuochen Wang, Anuradha Rout, Rayhan Habib Jibon, Qiang Wu, Yuliya Semenova

**Affiliations:** 1 Photonics Research Centre, School of Electrical and Electronic Engineering, Technological University Dublin, Dublin, Ireland; 2 Tyndall National Institute, University College Cork, Lee Maltings, Dyke Parade, Cork, Ireland; 3 Department of Mathematics, Physics and Electrical Engineering, Northumbria University, Newcastle Upon Tyne, NE1 8ST UK

**Keywords:** Fibre optics and optical communications, Liquid crystals

## Abstract

Microfluidic flowmeters are a powerful and highly accurate tool, enabling precise monitoring and measurements of flows of gases and fluids in a range of applications. Here we proposed and experimentally demonstrated a whispering gallery modes flowmeter composed of a liquid crystal-filled nested capillary. Whispering gallery modes are excited by a tapered fiber coupled perpendicularly to the nested capillary. The air flowing through the capillary cools it down, which leads to a temperature-induced change of the refractive index of the nematic liquid crystals. This change in turn leads to a spectral shift of the whispering gallery modes resonances, which can be linked to the airflow rate in the capillary. The temperature change in the liquid crystals was simulated considering the heat transfer between the liquid crystals and airflow in the capillary, which indicated that the liquid crystals temperature decreases in a nonlinear manner with the increase of the airflow rate. A flowmeter with the maximum sensitivity of 0.3423 nm·min·mL^−1^ in the flowrate range from 0 to 2.52 nm·min·mL^−1^ and a resolution of 5.72 pm was demonstrated in our experiment. The proposed sensor provides a platform for whispering gallery modes flowmeters and offers the advantages of good stability, high sensitivity, and miniature size.

## Abstract (web summary)

Wang and colleagues design a flowmeter composed of a liquid crystal-filled nested capillary which demonstrates thermal stability and high sensitivity. The device measures air flow via its cooling effect resulting in a measurable spectrum shift in whispering gallery modes.

## Introduction

With the development of microfabrication technologies, microfluidic devices and sensors have been extensively studied for applications in biological sensing^1^, chemical reactions detection^2^ and particle screening^3^. Microfluidic platforms require small sample volumes allowing to greatly decrease waste of the analytes, which makes it attractive for many applications including air or liquid metering. In recent years, microfluidic flowmeters integrated with fiber optics have attracted a lot of attention and several structures have been proposed and demonstrated. Yan et al. presented a microfluidic flowmeter by wrapping a microfiber coupler around a gold film coated capillary^4^. Liu et al. proposed a stacked capillary flowmeter sensor based on a microfiber Bragg grating (FBG) connected with a Co^2+^-doped optical fiber^5^. The liquid flow in the capillary was used to adjust the temperature of the stacked FBG. However, the complexity of fabrication of the device led to multiple challenges in its application in practice.

Due to the advantages of high sensitivity and small volume, whispering gallery modes (WGMs) resonator-based flowmeters have been extensively studied and a variety of flow sensors have been proposed and demonstrated. For example, a capillary flowmeter integrated with a dye-doped polymer with a sensitivity of 17 pm·sccm^−1^ has been reported in reference^6^. The shift in the laser peak wavelength reflected the flow rate due to temperature variations caused by the air flow. Another flowmeter utilized cooling effect of the gas flow within an Er:Yb- doped capillary resulting in the laser wavelength shift towards shorter wavelength^7^. However, a laser source had to be used to trigger the WGM laser, and a relatively high cost of the instrument potentially limited its practical applications.

Liquid crystal (LC) is a kind of material whose elongated rod-like molecules exhibit long-range orientational order while maintaining the mobility of conventional liquids. As a result of this, optical properties of LCs can be tuned by changing the alignment of the molecules under the influence of temperature or external electric field, which facilitates their wide range of applications in displays^8^, filters^9^, and sensing^10^. LCs-based fiber sensors in particular have attracted significant interest and various types of sensor configurations have been reported in literature. A dye-doped LC droplet WGM sensor was developed by Rui et al.^11^, where stearic acid-doped 4-cyano-4′-pentylbiphenyl microdroplet served as a platform for real-time and quantitative detection of urea under the illumination of a 532-nm laser. A temperature sensing scheme using LCs-filled Fabry-Perot cavity was proposed and demonstrated in^12^, taking advantage of the refractive index (RI) change of the LC under the influence of temperature and the Vernier effect due to the birefringence of the LC. Owing to its high RI and thermo-optics coefficients, LCs are ideal materials for integration with high sensitivity fiber sensors.

In this paper, we propose and demonstrate a flowmeter structure based on nested cylindrical WGM resonators, with the larger capillary filled with a nematic LC, and the WGM modes are excited using a tapered fiber placed perpendicularly to the larger capillary. The device was fabricated by tapering two nested capillaries with different diameters using the microheater brushing technique^13^. The flowmeter function was realized due to the change in the RI of the LC filling the larger capillary, caused by the air flow through the inner smaller capillary. The proposed scheme does not require the use of high-power lasers and highly complex fabrication. To the best of our knowledge, this is the first attempt to utilize a highly sensitive temperature response of a LC to realize a flow sensor. The proposed scheme shows the advantages of high sensitivity and miniature size, with many potential practical applications.

## Methods

### Fabrication of the LC-filled WGM capillary resonator

The nested thin-wall capillary structure was fabricated by tapering two capillaries with different diameters using the customized microheater brushing technique^13^. In our experiment, the polymer coating layers of commercial silica capillaries (Polymicro Technology) with the outer/inner diameters of 435 μm/320 μm and 850 μm/700 μm were both stripped off and then a short section (15 cm) of the smaller stripped capillary with the outer/inner diameters of 435 μm and 320 μm was inserted into the capillary with the larger outer/inner diameters of 850 μm and 700 μm to form the nested capillary structure. The two ends of the nested capillary structure were then fixed on the computer-controlled translation stages and the centre part was placed in the slit of the ceramic microheater (CMH-7019, NTT-AT) where the temperature was set to ~1300 °C. The customized computer program controlled the motion of the translation stages to ensure that the center of the nested capillary structure was heated and stretched simultaneously to achieve the final waist diameters of 60 μm and 30 μm for the outer and inner capillaries, respectively. The fabricated nested capillary structure was then fixed on a glass substrate using two droplets of a UV glue. This was realized with the help of two additional spacers, glued to the substrate, to ensure that the middle part of the capillary was suspended above the glass to minimize optical losses.

Next, a tapered fiber with a uniform waist diameter of ~1 μm was prepared using the same microheater brushing setup. The two ends of the tapered fiber were connected to the broadband light source (BBS, Thorlabs, S5FC1005S(P), 1500–1600 nm, diode current = 600 mA, full width at half maximum (FWHM) = 50 nm) through a polarization controller and the optical spectrum analyzer (OSA, Advantest, Q8384). For light coupling, the middle part of the nested capillary suspended over a glass substrate on a translation stage was placed in contact with the uniform waist region of the tapered fiber. Then, the nested capillary was gradually moved along the fiber taper axis until the WGM spectrum with the highest possible quality (Q)-factor was observed.

Two droplets of a UV glue were applied to the substrate to fix the relative positions of the tapered fiber and nested capillary. Finally, the empty volume between the outer and inner capillaries was filled with a nematic liquid crystal (MDA-05-2782, *n*_e_ = 1.6152, *n*_o_ = 1.4912, measured at 589.3 nm and 20 °C, and clearing point at 106 °C) (Licristal, Merck) using a syringe from one end of the nested capillary. After the LC was filled in the nested capillary, the capillary was disconnected from the syringe, and its ends were sealed with a UV glue.

### Experimental setup for experimental characterization of the flowmeter

**Fig. 1:**
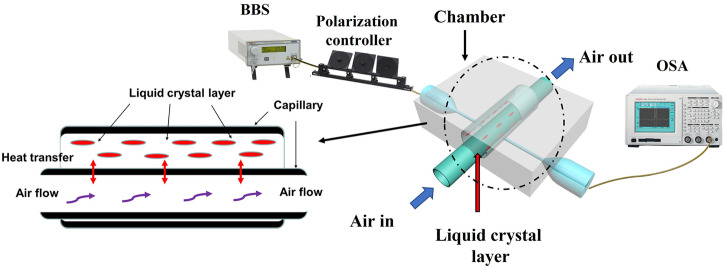
Schematic diagrams of the nested capillary-based flow sensor and experimental setup for its characterization. Light from BBS was pumped into the tapered fiber and coupled with the nested capillary after passing through the polarization controller. The transmission spectra from the tapered fiber were collected using an OSA. The tested sample was placed in a temperature insulated chamber to ensure the constant temperature. (BBS: broadband light source, OSA: optical spectrum analyzer).

The schematic diagram of the proposed flow sensor is shown in Fig. 1. One end of the inner capillary was connected to an air pump (YZ1515, Baoding Qili constant Pump, Co. Ltd) through a long tube, which provides the air flow with different flow rates (0–6.8) in standard cubic centimeters per minute (sccm) or (mL·min^−1^). It should be noted that the coupled WGM resonator was placed into a temperature insulated chamber to ensure the temperature stability. The pumped air goes directly through the capillary and exits into the surrounding. To ensure the accuracy of detection, the data collection at each flow rate commenced after the corresponding air flow has been applied through the capillary for 3 min.

## Results and discussion

### WGM spectra of the microcapillary resonator

**Fig. 2:**
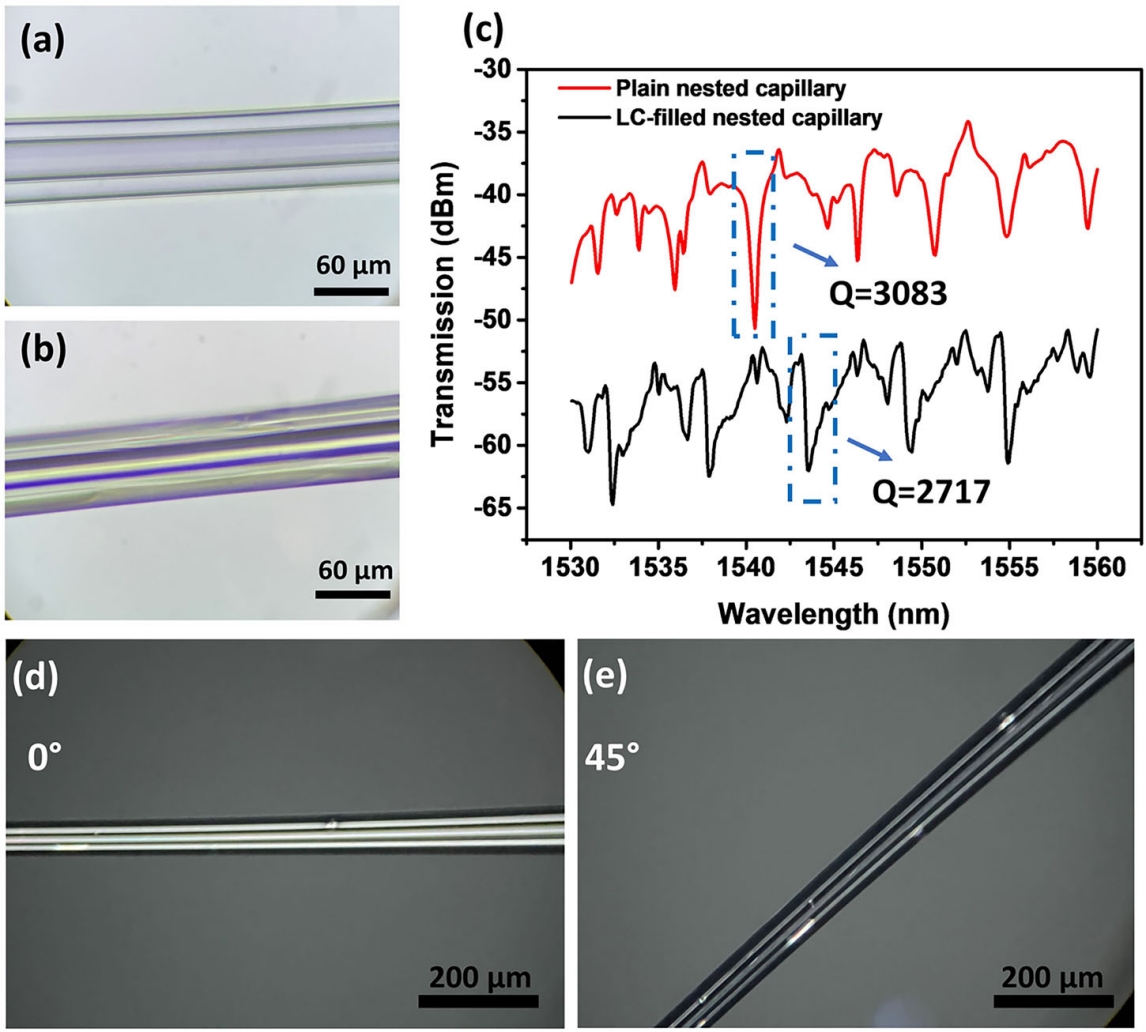
Characterization of the WGM nested capillary resonator. Microscopic images of the empty (**a**) and LC-filled (**b**) nested capillaries, (**c**) transmission spectra of the empty and the LC-filled nested capillaries, (**d**, **e**) polarization micrographs of the nested capillary infiltrated with MDA-05-2782 taken at different rotation angles. (WGM: whispering gallery mode, LC: liquid crystal).

Figure 2a, b show microscopic images of the fabricated nested capillary structure and the LC-filled nested capillary respectively. As one can see, the blurring effect arising from the scattering on the LC molecules can be observed within the LC-filled area. The transmission spectra of the empty and LC-filled capillaries are shown in Fig. 2c. As can be seen from the graphs, additional side-lobes appear in the transmission spectrum of LC-filled nested capillary due to the birefringence of the LC. The Q factors for the two transmission spectra were calculated as 3083 and 2717 using the resonant dips at 1540.47 nm and 1543.66 nm, and the corresponding FWHMs were measured as 0.50 nm and 0.57 nm, respectively.

To confirm the LC infiltration, the nested capillary was placed between crossed polarizers under a microscope. As can be seen from Fig. 2d, e, the light extinction phenomenon when rotating the capillary indicates that the LC has been successfully infiltrated in the nested capillary.

### Theoretical analysis and simulations

#### Analysis of light coupling conditions

**Fig. 3:**
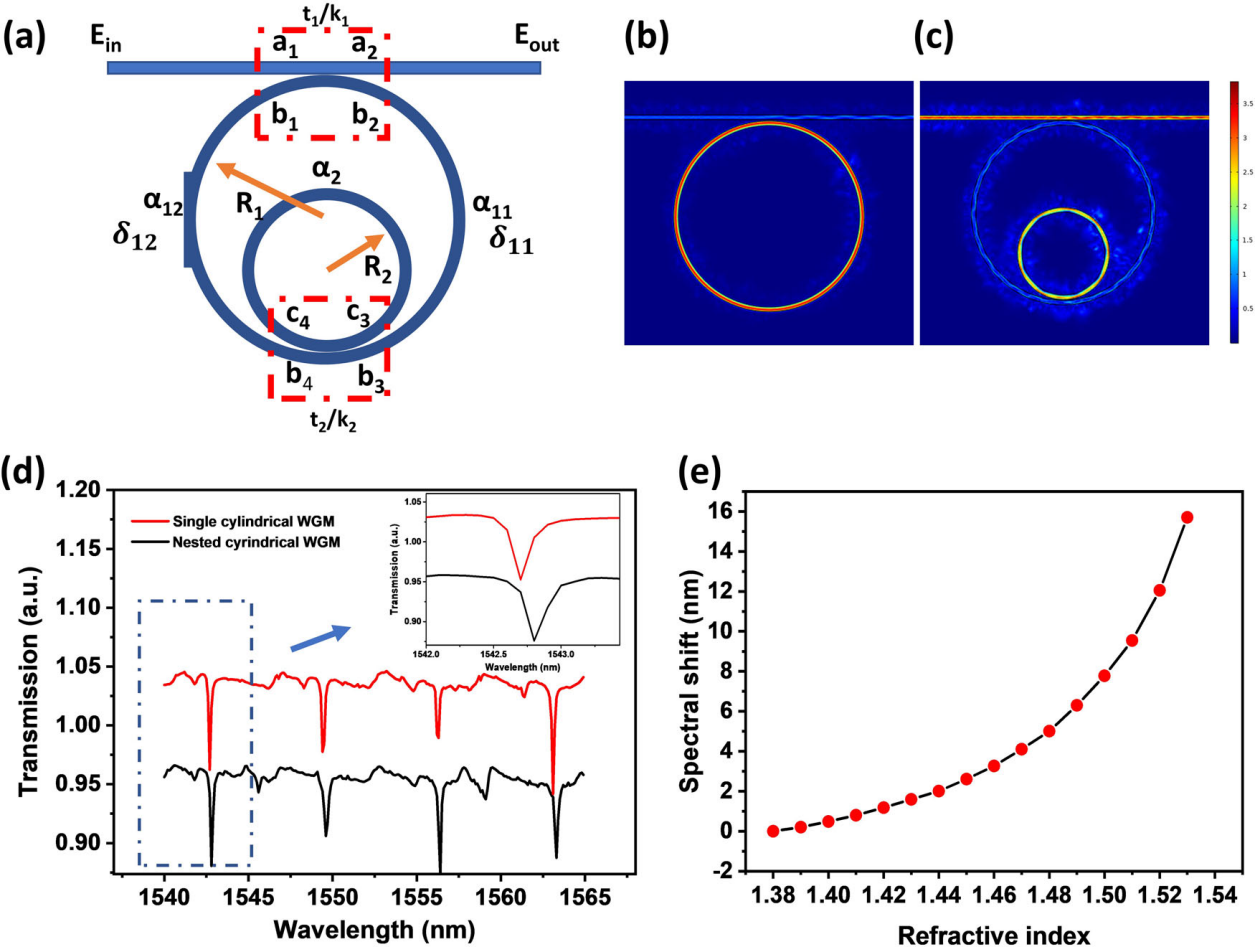
Simulation of the nested capillary coupling. **a** Schematic diagram of the nested cylindrical resonator and coupling fiber, simulated electric field intensity distributions in (**b**) single cylindrical resonator and **c** nested cylindrical resonator structure, **d** simulated transmission spectra for the single cylindrical and the nested cylindrical resonators as well as the zoomed graph in a wavelength range from 1540 to 1545 nm, **e** selected transmission dip shift as a function of the liquid crystal RI. (WGM: whispering gallery mode).

The light coupling between a nested capillary and a tapered fiber can be analysed based on the transfer matrix method in nested rings^14^. The schematic of the coupling system is shown in Fig. 3a. When a nested capillary is perpendicularly coupled with a tapered fiber, the interactions in the system can be described as:

1 $$\left[\begin{array}{c}{a}_{2}\\ {b}_{2}\end{array}\right]=\left[\begin{array}{cc}{t}_{1} & {{{{{\rm{j}}}}}}{k}_{1}\\ {{{{{\rm{j}}}}}}{k}_{1} & {t}_{1}\end{array}\right]\left[\begin{array}{c}{a}_{1}\\ {b}_{1}\end{array}\right]$$

2 $$\left[\begin{array}{c}{b}_{4}\\ {c}_{4}\end{array}\right]=\left[\begin{array}{cc}{t}_{2} & {{{{{\rm{j}}}}}}{k}_{2}\\ {{{{{\rm{j}}}}}}{k}_{2} & {t}_{2}\end{array}\right]\left[\begin{array}{c}{b}_{3}\\ {c}_{3}\end{array}\right]$$

where ${t}_{1}$ and ${t}_{2}$ represent the transmission coefficients of the nested rings, ${k}_{1}$ and ${k}_{2}$ represent the coupling coefficients from the tapered fiber to the outer ring, and from outer ring resonator to the inner ring resonator. ${a}_{i}$ and ${b}_{i}$ are the electric fields in the coupling regions shown in Fig. 3a. ${t}_{i}^{2}+{k}_{i}^{2}=1$ is true if the coupling and transmission losses in the coupling area are neglected.

The light propagation in a resonator can be written as: ${b}_{3}={\alpha }_{11}{{{{{{\rm{e}}}}}}}^{{{{{{\rm{j}}}}}}{\delta }_{11}}{b}_{2}$, ${b}_{1}={\alpha }_{12}{{{{{{\rm{e}}}}}}}^{{{{{{\rm{j}}}}}}{\delta }_{12}}{b}_{4}$, ${c}_{3}={\alpha }_{2}{{{{{{\rm{e}}}}}}}^{{{{{{\rm{j}}}}}}{\delta }_{2}}{c}_{4}$, where ${\alpha }_{i}$ and ${\delta }_{i}$ are the loss coefficient and phase shift in the corresponding propagation path within the resonator. ${\delta }_{i}$ can be written as ${\delta }_{i}=\frac{4{{{{{{\rm{\pi }}}}}}}^{2}{R}_{i}{n}_{{{{{{\rm{eff}}}}}}}}{\lambda }$, where ${R}_{i}$ is the radius of the cylindrical resonator, ${n}_{{{{{{\rm{eff}}}}}}}$ is the RI of the selected WGM mode, $\lambda$ is the resonant wavelength. According to the transfer matrix and the formulas for light propagation, $\frac{{b}_{4}}{{b}_{3}}$ is described as:

3 $$\frac{{b}_{4}}{{b}_{3}}=\frac{{t}_{2}-{\alpha }_{2}{{{{{{\rm{e}}}}}}}^{{{{{{\rm{j}}}}}}{\delta }_{3}}}{1-{\alpha }_{2}{t}_{2}{{{{{{\rm{e}}}}}}}^{{{{{{{\rm{j}}}}}}\delta }_{3}}}=I$$

The final transmission can be written as:

4 $$\frac{{E}_{{out}}}{{E}_{{in}}}=\frac{{a}_{2}}{{a}_{1}}=\frac{{t}_{1}-{\alpha }_{1}{{{{{{\rm{e}}}}}}}^{{{{{{\rm{j}}}}}}{\delta }_{1}}I}{1-{t}_{1}{\alpha }_{1}{{{{{{\rm{e}}}}}}}^{{{{{{\rm{j}}}}}}{\delta }_{1}}I}$$

The simulations of the electric field distribution in the above nested capillary resonator were carried out using COMSOL Multiphysics (5.5) software in 2-dimensional view. In the simulation, the diameters for the outer and inner cylindrical resonators are set as 80 μm and 40 μm, respectively. The wall thickness for both the capillaries is set to 2 μm. A silica fiber taper with a uniform waist diameter of 2 μm is assumed as the light coupling taper. It is assumed that a Gaussian beam with the cross-section of 1 μm is pumped into a fiber from the input port (on the left side of the diagram). It is assumed that the nested capillary structure is empty and surrounded by air (RI is equal to 1). The material of the nested cylindrical structure is silica. The electric field distributions for the single cylindrical and nested cylindrical resonators at the wavelengths of 1556.2 nm and 1559.3 nm are shown in Fig. 3b, c. Figure 3d illustrates the simulated transmission spectra of the coupling fiber taper for the single cylindrical and nested cylindrical resonators in the wavelength range from 1540 nm to 1565 nm. It can be seen from the graphs that introduction of the inner cylindrical resonator coupled with the outer cylinder results in the appearance of new resonant dips around 1546 nm and 1559.3 nm. The zoomed graph in Fig. 3d illustrates a red shift of 0.25 nm in the transmission spectrum due to the introduction of the inner capillary.

As the next step we investigated how the RI of the LC influences the transmission spectrum of the nested cylindrical resonator. The n_e_ and n_o_ for MDA-05-2782 at 1550 nm can be approximately estimated as 1.5769 and 1.4623 using the three-coefficient Cauchy model^15^. Thus the average refractive index for the LC can be calculated as 1.5005 using the equation: *n*_average_ = (*n*_e_ + 2*n_o_)/3^15^. In our simulation, the surrounding area for the inner capillary refers to the LC-filled space between the two capillaries. Since the operating principle of our sensor relies on temperature induced changes of the liquid crystal’s RI and considering a typical thermo-optic coefficient of a nematic LC as −9 × 10^−4^ per °C^16^, a temperature change in the range of ±10 °C, should result in changes of the LC’s RI in the range from 1.4915 to 1.5095.

Figure 3e shows the simulated dependency of the spectral shift of the selected resonant dip at 1546 nm versus the surrounding RI in the range of RIs from 1.38 to 1.53. As one can see from the plot, the WGM resonance red shifts with the increase of the RI of the LC within the nested capillary. A greater spectral shift can be observed at higher RI values, especially when the RI is >1.48.

#### Simulation of the heat transfer

**Fig. 4:**
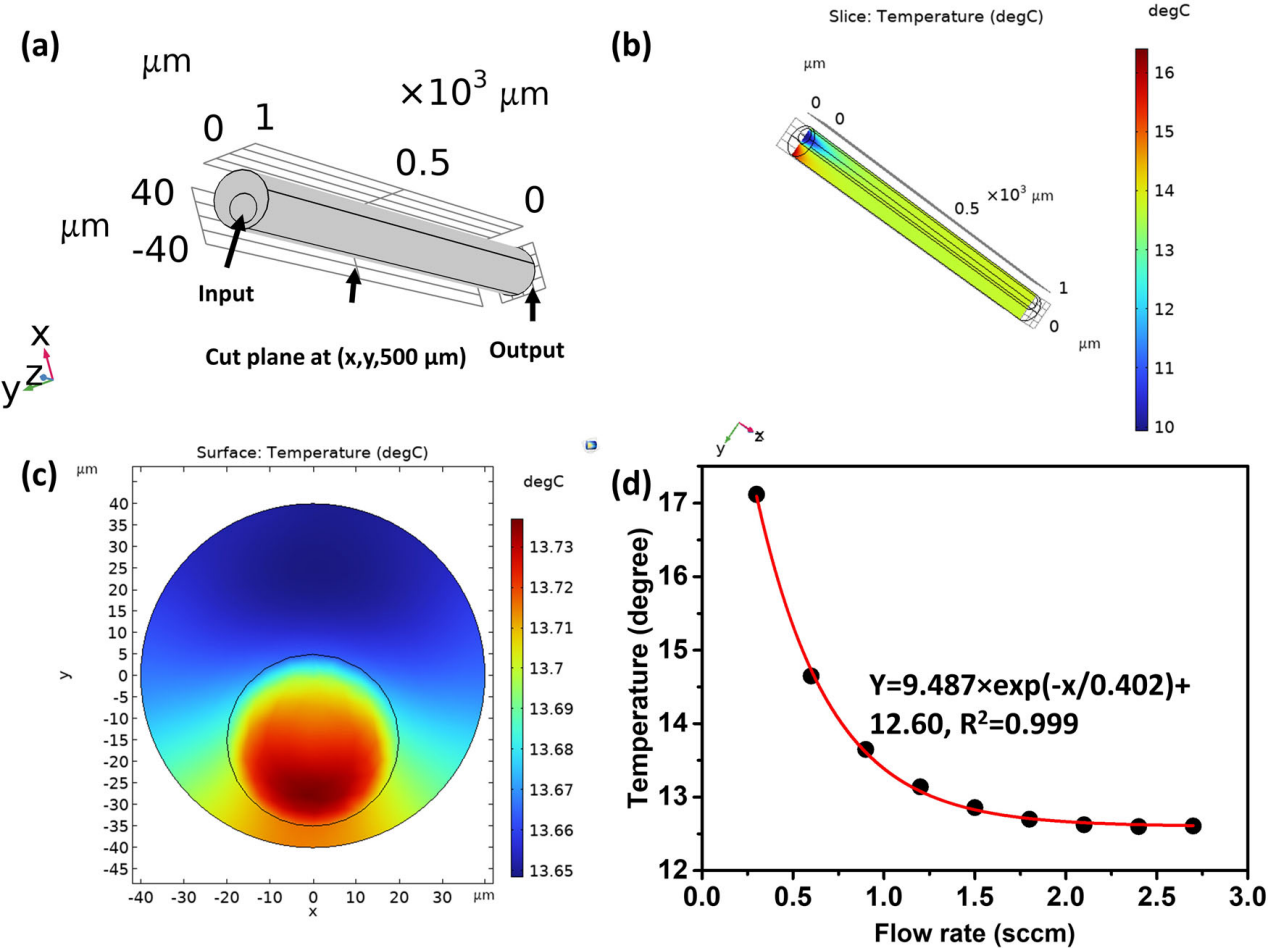
Simulation of the heat transfer in the nested capillary. **a** Schematic diagram of the nested capillary structure, **b** 3-dimensional temperature distribution within the nested capillary at the air flow rate of 0.9 sccm, **c** temperature distribution within the x–y cross section at the z coordinate of 500 µm, **d** minimal temperature of the LC area in figure (**c**) as a function of the air flow rate. (LC: liquid crystal).

The heat transfer in the nested capillary was simulated using a 3-dimensional model in COMSOL. The choice of a suitable study method for laminar or turbulent flows in the simulation is determined by the Reynolds number, which can be calculated as^17^:

5 $${{{{\mathrm{Re}}}}}=\frac{\rho {vL}}{\mu },\left\{\begin{array}{c}\le \!2300\,{{{{{\rm{Laminar}}}}}}\,{{{{{\rm{flow}}}}}}\hfill\\ > 2300\,{{{{{\rm{Turbulent}}}}}}\,{{{{{\rm{flow}}}}}}\end{array}\right.$$

where *ρ* and *v* are the density and velocity of the air, *L* is the length of the capillary, µ is the dynamic viscosity of the air. The laminar flow method was implemented to simulate the heat distribution in the nested capillary due to the small value of the Reynolds number (574) in our case. In the simulation, the geometry of the model was following the structure of the nested capillary, where the diameters of the two cylinders are 80 µm and 40 µm respectively, as shown in Fig. 4a. In COMSOL, the silica capillary wall with 2 µm thickness was assumed as a thin layer to simplify the calculation process. The LC layer is assumed to fill the space between the two cylinders with the length of 1000 µm and the air flows in from the input port shown in Fig. 4a. In the simulation the values for the thermal conductivity and heat capacity were assumed to be 3400 J·kg^−1^·K^−1^ and 0.17 W·m^−1^·K^−1^ respectively, corresponding to those of octyl cyanobiphenyl (8CB)^18^, which should be reasonably close to those for the material used in our experiments (MDA-05-2782). The density of the LC was assumed as 985 kg·m^−3^. In the simulation, the temperatures of the LC and the air were assumed as 27 °C and 10 °C, respectively for the full demonstration of the heat transfer process. Figure 4b illustrates the temperature distribution in the nested capillary within y–z section at an x coordinate of 0 µm at the flow rate of 0.9 sccm. As one can see from the graph, the temperature of the LC near the input port drops significantly to ~16 °C due to the heat transfer between the air and LC because of the continuous airflow through the inner capillary. With the increase of the z coordinate, the temperatures of the air and LC are stationary near 14 °C, indicating the occurrence of the thermal equilibrium. The tapered fiber is placed perpendicularly to the capillary to facilitate light coupling, and most of the light field energy is focused inside of the wall and the inner surface of the capillary near the coupling area^13^. Thus, the temperature distribution within the capillary waist at this position would significantly influence the performance of the WGM sensor in our case, corresponding to the region around 500 µm along the z-coordinate. The temperature distribution within the x–y cross-section at z coordinate of 500 µm is illustrated in Fig. 4c. As can be seen from the graph, the heat transfer leads to the temperature decrease of the LC from 27 °C to around 13.6 °C despite the existence of the silica boundary between the LC and the air. The temperature difference between the air and the LC is <0.1 °C, which can be explained by the thermal equilibrium during the heat transfer process. Figure 4d shows the dependency of the minimum temperature of the LC at the cross-section plane (x, y, 500 µm) as a function of the air flow rate. As one can see, the increase of the flow rate from 0.3 sccm to 1.2 sccm results in a significant decrease of the temperature of the LC which then saturates as the flow rate reaches 1.5 sccm. The tendency of the temperature with respect to the flowrate can be fitted as $y=9.487\times \exp \left(-\frac{x}{0.402}\right)+12.6$ with the R^2^ of 0.999. The maximum temperature sensitivity of the sensor is 4.31 °C·sccm^−1^.

### Study of the influence of the flow rate on the WGM spectrum

**Fig. 5:**
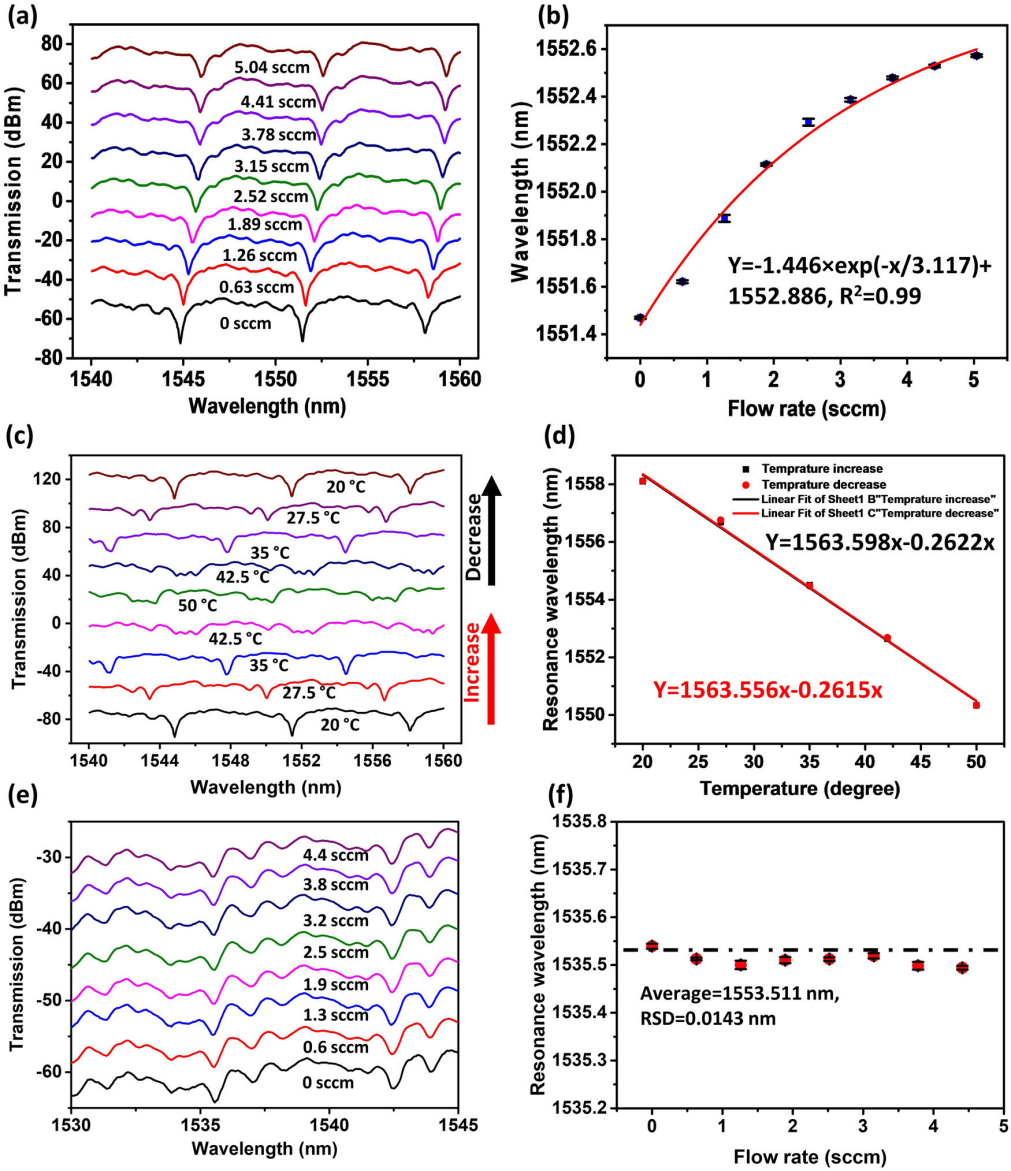
Spectral characterization of the nested capillary resonator. **a** Measured WGM spectra of the LC filled nested capillary structure at different air flow rates, **b** selected spectral dip at 1551.5 nm versus the flow rate, **c** WGM spectra of the nested capillary resonator measured at different temperatures, **d** linear fitting of the spectral shift data versus temperature for the selected dip at 1551.6 nm in figure (**c**), **e** experimental WGM spectra of the nested capillary resonator without LC filling, **f** selected spectral dip versus the flow rate. (WGM: whispering gallery mode, LC: liquid crystal, sccm: nm·min·mL^−1^). (Note: Error bars were calculated using standard deviation of measurements for five times).

Figure 5a shows the experimental transmission spectra of the nested capillary resonator corresponding to different air flow rates. Figure 5b illustrates the dependency of the selected resonant dip position (at circa 1551.5 nm) versus the flow rate and its exponential fit in the range of flow rates from 0 to 5.04 sccm. As can be seen from the graph, the selected WGM resonance wavelength increases in a non-linear fashion with the increase of the flow rate. The maximum sensitivity of the proposed sensor was calculated as 0.3423 nm·sccm^−1^ in the range of flow rates from 0 to 2.52 sccm. The maximum experimentally measured spectral shift is 1.1 nm corresponding to the increase of the flow rate from 0 to 5.04 sccm.

The experimentally observed spectral shift of the WGM resonances is due to changes in the effective RI of the liquid crystal caused by the heat transfer between LC and the airflow.

The temperature-induced wavelength shift can be expressed using the following equation^19^:

6 $$\Delta \lambda =\lambda \left(\alpha \Delta T+\frac{1}{{n}_{{{{{{\rm{core}}}}}}}}\frac{d{n}_{{{{{{\rm{core}}}}}}}}{{dT}}\Delta T+\frac{1}{{n}_{{{{{{\rm{wall}}}}}}}}\frac{d{n}_{{{{{{\rm{wall}}}}}}}}{{dT}}\Delta T \right)$$

where $\alpha$ is the thermal expansion coefficient of the cylindrical WGM resonator, $\frac{d{n}_{{{{{{\rm{core}}}}}}}}{{dT}}$ and $\frac{d{n}_{{{{{{\rm{wall}}}}}}}}{{dT}}$ are the thermo-optic coefficients of the nematic LC and the silica capillary wall, ${{{{{{\rm{n}}}}}}}_{{{{{{\rm{core}}}}}}}$ and ${{{{{{\rm{n}}}}}}}_{{{{{{\rm{wall}}}}}}}$ are the effective RIs of the nematic LC and silica capillary, respectively. $\Delta T$ is the change in temperature due to the heat transfer between the LC and the airflow. $\lambda \frac{1}{{n}_{{{{{{\rm{core}}}}}}}}\frac{{{{{{\rm{d}}}}}}{n}_{{{{{{\rm{core}}}}}}}}{{dT}}\Delta T$ is the main factor contributing to the spectral shift since the values of $\alpha$ and $\frac{d{n}_{{{{{{\rm{wall}}}}}}}}{{dT}}$ are negligibly small^20^. Owing to the negative $\frac{d{n}_{{{{{{\rm{core}}}}}}}}{{dT}}$ of the LC^16^, the resonant wavelength shifts towards longer wavelengths when the temperature of the LC decreases due to the airflow through the inner capillary, which is in agreement with the experimental results in Fig. 5a, b.

The spectral position of a WGM resonance can be described approximately by the following equation^21^:

7 $${\lambda }_{l} \, \approx \, \frac{2\pi a}{l}{n}_{{{{{{\rm{eff}}}}}}}$$

where *λ*_*l*_, is an *l*-th order resonance, *a* is the outer radius of the WGM resonator, ${n}_{{{{{{\rm{eff}}}}}}}$ is the effective refractive index of the mode. Considering the structure of the LC-filled microcapillary, ${n}_{{{{{{\rm{eff}}}}}}}$ can be expressed as^22^:

8 $${n}_{{{{{{\rm{eff}}}}}}}={f}_{1}{n}_{1}+{f}_{2}{n}_{2}+{f}_{3}{n}_{3}$$

where ${n}_{1}$, ${n}_{2}$, and ${n}_{3}$ are the RIs of the capillary core, capillary wall, and the surrounding air, respectively and *f*_*1*_, *f*_*2*_ and *f*_*3*_ are the corresponding fractions of the mode energy in each layer.

The fractions of the mode energy *f*_1_ (inside the capillary), *f*_2_ (capillary wall), and *f*_3_ (outside the capillary) can be calculated numerically from the corresponding mode field energy distributions (${I}_{1},{I}_{2}$ and ${I}_{3}$). The values of ${I}_{1},{I}_{2}$ and ${I}_{3}$ are calculated from^23^:

9 $${I}_{1}={{\int }_{\!\!\!0}^{b}}{{{n}_{1}}^{2}{k}^{2}r\left[{A}_{l}{J}_{l}\left({n}_{1}{kr}\right)\right]}^{2}{dr}$$

10 $${I}_{2}={{\int }_{\!\!\!b}^{a}}{{n}_{2}}^{2}{k}^{2}r{\left[{B}_{l}{H}_{l}^{\left(2\right)}({n}_{2}{kr})+{H}_{l}^{\left(1\right)}\left({n}_{2}{kr}\right)\right]}^{2}{dr}$$

11 $${I}_{3}={{}{\int }_{\!\!\!a}^{\infty }}{{n}_{3}}^{2}{k}^{2}r{\left[{D}_{l}{H}_{l}^{\left(1\right)}\left({n}_{3}{kr}\right)\right]}^{2}{dr}$$

where *r* is the radial direction within the capillary, *a* and *b* are the outer and inner radii of the capillary, ${J}_{{{{{{\boldsymbol{l}}}}}}}$ is a cylindrical Bessel function of the first kind with order *l*, ${H}_{l}^{\left(1\right)}$ and ${H}_{l}^{\left(2\right)}$ are cylindrical Hankel functions with order *l*, *k* is the wavevector in vacuum, ${A}_{l}$, ${B}_{l}$, and ${D}_{l}$ are constants, *n*_*1*_, *n*_*2*_, and *n*_*3*_ are the RIs of the capillary core, capillary wall (silica) and the surrounding air, respectively. For the liquid crystal-filled capillary, the increase of ${n}_{{{{{{\rm{core}}}}}}}$ leads to the increase of the fraction ${f}_{1}=\frac{{I}_{1}}{{I}_{1}+{I}_{2}+{I}_{3}}$, and decrease of the fractions ${f}_{2}=\frac{{I}_{2}}{{I}_{1}+{I}_{2}+{I}_{3}}$, and ${f}_{3}=\frac{{I}_{3}}{{I}_{1}+{I}_{2}+{I}_{3}}$, thus resulting in a change of the ${{{{{{\rm{n}}}}}}}_{{{{{{\rm{eff}}}}}}}$ and a shift of the resonant wavelength as was demonstrated in reference^24^. Therefore, the temperature-induced change of the RI of the LC in the capillary core influences the value of ${{{{{{\rm{n}}}}}}}_{{{{{{\rm{eff}}}}}}}$, thus affecting the spectral positions of the WGM resonances of the capillary.

As can be seen from the experimental result in Fig. 5b, an increase in the airflow rate leads to an increase in the spectral shift of the selected resonance. This can be explained as follows. In the absence of the airflow through the capillary, its temperature remains constant, and there is no shift in the WGM spectrum. When the air passes through the inner capillary at a certain flow rate, heat transfer occurs between the air and LC, and the airflow cools the LC down to a certain temperature, leading to an increase of LC’s effective RI, and a corresponding red shift of the WGM resonances. An increase in the airflow rate increases the heat transfer rate between the air and LC within the capillary, resulting in a greater temperature change, larger increase in the effective RI of the LC, and a larger spectral shift of the resonances. It should be noted however that at higher flow rates the spectral wavelength shift of the WGM resonances shows the tendency to saturate.

As we demonstrated previously in our heat transfer simulation, the temperature of the LC decreases in a non-linear fashion with the increase of the flow rate and shows a slowdown trend at higher airflow rates through the capillary. Moreover, due to the non-linear nature of the temperature dependence of the LC’s RI^25^, at lower temperatures the same temperature change leads to a smaller change in the LC’s RI.

The results of our simulations and experiments also in a good agreement in terms of sensitivity of the sensor’s response to the air flow rate. Specifically, our heat transfer simulations presented in Fig. 4d demonstrated that an increase in the flow rate from 0 to 2.75 sccm results in the decrease of the minimum temperature of the liquid crystal by circa 4.5 °C. As the temperature of the LC decreases due to the cooling effect, the corresponding change in its RI can be estimated as follows. Given that the average RI of the liquid crystal was calculated as 1.5005 at 20 °C, assuming the LC’s thermo-optic coefficient as −9 × 10^−4^ per °C^16^, a 4.5 °C temperature drop should result in an increase of the average RI to 1.5046. From the simulated dependence of the wavelength shift of the WGM resonance presented in Fig. 3e, the wavelength shift corresponding to the change of RI from 1.5005 to 1.5046, should lead to the spectral red shift of the resonance of about 0.714 nm. This result is in a good agreement with our experiment. As one can see form Fig. 5b, an increase in the flow rate from 0 to 2.75 sccm leads to the red shift of the selected WGM dip of ~0.82 nm.

The smallest detected change in the air flow rate, or the detection limit (DL), for the proposed scheme can be determined as^26^:

12 $${DL}=\frac{R}{S}$$

where R and S are the sensor’s resolution and sensitivity, respectively. R can be calculated as^26^:

13 $$R=3\times \sqrt{{\sigma }_{{{{{{\rm{ampl}}}}}}-{{{{{\rm{noise}}}}}}}^{2}+{\sigma }_{{{{{{\rm{temp}}}}}}-{{{{{\rm{induced}}}}}}}^{2}+{\sigma }_{{{{{{\rm{spect}}}}}}-{{{{{\rm{res}}}}}}}^{2}}$$

where ${\sigma }_{{{{{{\rm{ampl}}}}}}-{{{{{\rm{noise}}}}}}}$, ${\sigma }_{{{{{{\rm{temp}}}}}}-{{{{{\rm{induced}}}}}}}$, ${\sigma }_{{{{{{\rm{spect}}}}}}-{{{{{\rm{res}}}}}}}$ represent the amplitude noise variance, temperature-induced noise, and spectral resolution of the equipment, respectively. ${\sigma }_{{{{{{\rm{ampl}}}}}}-{{{{{\rm{noise}}}}}}}$ was calculated to be 1.9 pm according to the equation: ${\sigma }_{{{{{{\rm{ampl}}}}}}-{{{{{\rm{noise}}}}}}}=\frac{\Delta \lambda }{4.5({{SNR}}^{0.25})}$^26^, where $\Delta \lambda$ is the FWHM of the resonance dip, which relates to the resonator’s Q factor, SNR is the signal-to-noise of the system, which was assumed as 60 dB in our case. ${\sigma }_{{{{{{\rm{ampl}}}}}}-{{{{{\rm{noise}}}}}}}$ was calculated as 1.9 pm. Noise variance associated with temperature fluctuations $({\sigma }_{{{{{{\rm{temp}}}}}}-{{{{{\rm{induced}}}}}}})$ was taken as 0.01 pm. The spectral resolution of the equipment in our experiment is 10 pm ± 3%, so that the position of the resonant mode is uniformly distributed between −0.3 pm and 0.3 pm, which leads to the value of 0.17 pm for ${\sigma }_{{{{{{\rm{spect}}}}}}-{{{{{\rm{res}}}}}}}$. R was calculated to be 5.72 pm and the DL was determined as 0.0167 sccm for the LC-filled nested capillary.

To better understand the heat transfer in the nested capillary and to establish the relationship between the airflow and the temperature change of the LC in the capillary, the temperature response of the nested capillary was characterized using transmission spectra at zero sccm flow rate and shown in Fig. 5c. The temperature of the LC was controlled by a hot stage placed below the nested capillary resonator. As one can see from Fig. 5c, the spectral dips in the transmission spectrum experience blue shifts with the temperature increase from 20 °C to 50 °C, while decreasing the temperature back to 20 °C causes red shifts of the spectral dips. Figure 5d illustrates the experimental data and linear fitting for the spectral dip (at circa 1551.5 nm) as a function of temperature, where the temperature sensitivity of the nested capillary was estimated as 0.2615 nm·°C^−1^. Based on the airflow response and the temperature response of the nested capillary, the relationship between the flowrate and temperature in the nested capillary was estimated as 1.31 °C·sccm^−1^. The temperature change in the nested capillary can be roughly estimated as 4.17 °C.

To establish the influence of the LC-filling on the proposed flow sensor, a similar experiment was carried out for the nested capillary structure in the absence of the LC material. Figure 5e shows the spectral response of the resonator at different flow rates in the range from 0 to 5.04 sccm. Figure 5f illustrates the selected wavelength resonance (near 1535.5 nm) at different flow rates of the pump for the nested capillary resonator filled with air. As can be seen from the plot, the dip wavelength experiences only minor fluctuations with the increase of the flow rate. The mean resonance wavelength was determined to be 1553.511 nm, with a relative standard deviation (RSD) of 0.0143 nm.

### Investigation of the dynamic response of the flowmeter

**Fig. 6:**
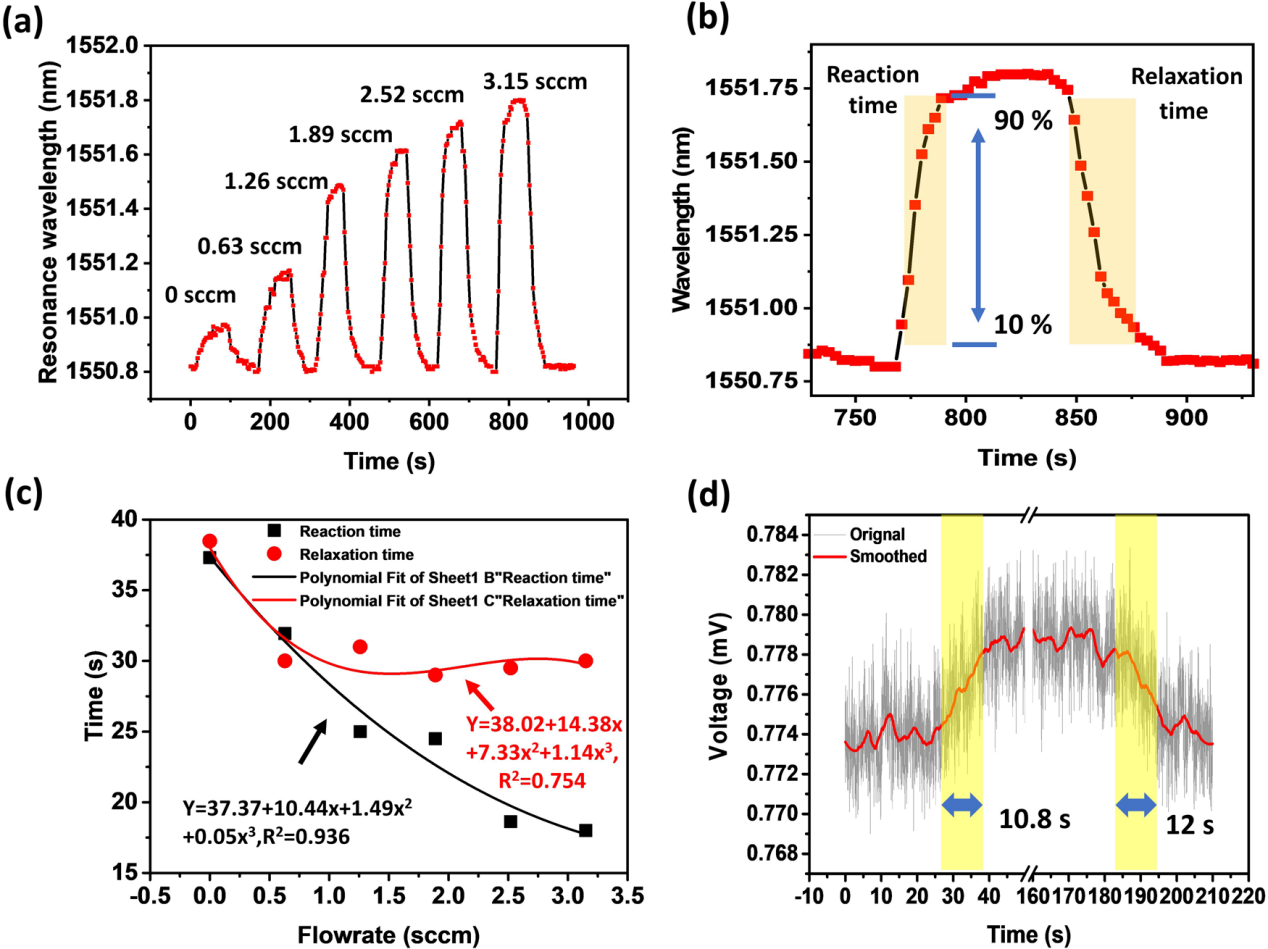
Investigation of the dynamic response of the flowmeter through the transmission spectrum. **a** Wavelength response of the nested capillary flowmeter at different flow rates, **b** zoomed-in plot of the figure (**a**) in a time period between 730th s and 925th s, **c** the reaction and relaxation times versus the flow rate, **d** time response of the proposed sensor corresponding to changes in the flow rate from 0 to 3.15 sccm, and then back to 0. (sccm: nm·min·mL^−1^).

To evaluate the dynamic response of the proposed flowmeter, similar experiments were carried out where the monitoring OSA was working in a quick scan mode. Figure 6a shows the spectral wavelength of the selected spectral dip (at circa 1551.8 nm) versus time for the nested capillary in a series of tests where various air flow rates were applied. As can be seen from the plots, abrupt wavelength shifts occurred when the air pump was switched on and off. Figure 6b illustrates the spectral response of the nested capillary in a time period between 730^th^ s and 925^th^ s. The response time is defined as the time corresponding to 10–90% of its maximum spectral shift of the resonance when the air pump is switched on or off ^27^. Figure 6c illustrates the proposed sensor’s reaction and relaxation times at different flow rates. As one can see, the reaction time decreases as the flow rate of air increases, while the relaxation time for the proposed sensor decreases significantly at first and is followed by small fluctuations around 30 s. The reaction and relaxation times dependencies versus flowrate were fitted with polynomial functions as shown in Fig. 6c. The more significant time difference between reaction and relaxation time with the increase of the flow rate can be attributed to the slow thermal dissipation process of the nest capillary in an insulated box. The relatively short response times indicate good suitability of the proposed flowmeter for many potential applications. It should be noted that in our experiment, various airflows were applied to the nested capillary with interruptions of 100 s to minimize the influence of heat accumulation or dissipation during the previous step. Given the interruption time used in experiment, it is expected that the response times for proposed sensor should be similar for both progressive increase and progressive decrease of the airflow rate. It should be noted however, that the response time of the sensor is likely to be longer if a continuous airflow with no interruptions is applied with step changes of the flow rates, especially if the flowrates decrease. The latter is because when the airflow rate decreases, the liquid crystal material will need some time to gather the heat from its surroundings and return to original temperature.

To determine the response time of the proposed sensor more accurately, another experiment was carried out, where the OSA in the experimental setup shown in Fig. 1 was replaced by a photodetector (Thorlabs, PDA10CS-EC) connected to an oscilloscope (Keysight, MSO-X2022A). As can be seen from the Fig. 6d, the abrupt voltage changes at 30th and 184th seconds corresponding to switching the air flow on and off, demonstrate the average response time of the 11.4 s for the case of the flow rate of 3.15  sccm. It should be noted that the response time of the proposed sensor can be further improved by optimizing the structure of the sensor, such as the volume of the LC and heat exchange area to improve the efficiency of the heat transfer between the LC and the flow. Moreover, there is a wide range of applications where a second order response time is sufficient. One example of potential applications where the response time for a flowmeter is less critical is monitoring of injection pumps, which is essential to ensure proper patient treatment and reduce medication errors that can lead to severe injury or death. E.g., a microfluidic thermal flowmeter based on the heat transfer between the heater and liquid flow was demonstrated in reference^28^. The response time of the sensor was ~4 s for a 1.5 mm wide channel, allowing for monitoring the flowrate of the injection pump in real time.

### Stability tests and study of the temperature effect on sensing performance

**Fig. 7:**
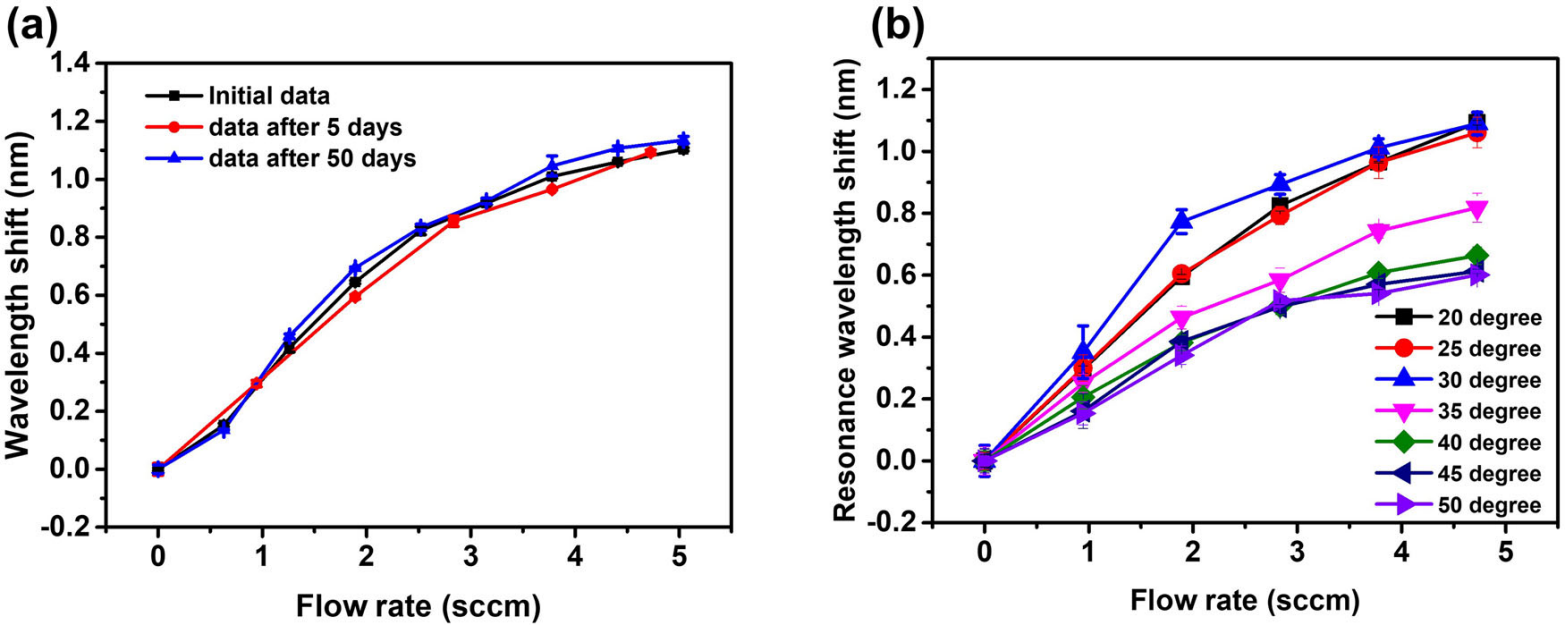
Spectral characterization of the flowmeter for the stability test and temperature effect investigation. **a** Spectral shift of the WGM resonance versus flow rate for the nested capillary flowmeter detected on the 1st, 5th, and 50th days, **b** spectral shift versus flow rate at different temperatures. (WGM whispering gallery mode, sccm nm·min·mL^−1^). (Note: Error bars were calculated using standard deviation of measurements for five times).

To explore the stability and repeatability of performance of the flowmeter, identical experiments were carried out with the same sample after 5 and then 50 days since the initial experiment. As one can see in Fig. 7a, the maximum shift of 1.1 nm was observed after 50 days since the initial experiment corresponding to <10% difference in the spectral shift, which demonstrates good stability and repeatability of performance. Given the temperature sensitivity of the nematic LC, the sensing performance of the flowmeter at different temperatures was characterized by placing the sensor on a hot stage of a temperature controller in an insulated box. The temperature was increased from 20 °C to 50 °C with intervals of 5 °C, and at each temperature the sensor was tested by increasing the flow rate with an interval of 0.945 sccm. Figure 7b shows the dependency of the spectral shift versus the flow rate for the proposed sensor at different temperatures. As can be seen from the graph, the maximum spectral shift remained stable, and the sensitivity of the sensor slightly raises with the increase of temperature in the range from 20 °C to 30 °C, which is likely due to a better heat transfer occurring at higher LC temperatures. It should be noted that further increase in the temperature from 30 °C to 60 °C leads to a deterioration of the sensor performance, which can be explained by the smaller RI of the chosen LC material at higher temperatures.

### Comparison and performance analysis of the different flowmeter technologies

Finally, we carried out a performance comparison of the proposed sensor with various flowmeters reported in the literature. The results of this comparison are summarized in Table 1. It should be noted that achieving a uniform unit conversion across all the reviewed flowmeters is challenging due to the specifics of the interrogation methods implemented in each case. As can be seen from the table, several technologies have been implemented to realize flowmeters for accurate monitoring and measurement of gas and liquid flows, including microelectromechanical (MEMS)^29^, piezoelectric^30^, electroconductive^31,32^ and optical^6,7,33–35^ sensors.

**Table 1:** Comparison of different flowmeter sensors

Type of sensor	Power consumption	Sensitivity		Range	Response time
CMOS MEMS^29^	<1.72 mW	1.15 V·s·m^−1^		−15–15 m·s^−1^	0.048 s
Piezoelectric Cantilever^30^	Not available	0.021 V·s·m^−1^		4.7–10.7 m·s^−1^	1 s
Reduced graphene oxide film^31^	Not available	32–50% in 7.2 m·s^−1^		0.026 m·s^−1^–7.2 m·s^−1^	64 s
GaN Chip with a PDMS membrane^32^	12.2 mW LED	0.266 µA·s·m^−1^		0–50 m·s^−1^	0.012 s
Hot-wire FBG^33^	Pump laser:1480 nm 0–530 mW BBS:1550 nm	28.60 µW·s·m^−1^		0–11 m·s^−1^	0.3 s
Silver film coated FBG^34^	228 mW BBS, 1520–1620 nm	6.6 × 10^−3 ^pm·min·mL^−1^		0–5.3 × 10^5 ^mL·min^−1^	Not available
Fabry-Perot Interferometer^35^	5 mW BBS:1530–1590 nm	3130 pm·s·m^−1^		0–7 m·s^−1^	0.4 s
Dye-doped polymer WGM laser^6^	274.1 μJ·cm^−2^, 1030 nm Yb:YAG pulse laser	17 pm·min·mL^−1^		0–50 mL·min^−1^	Not available
Er:Yb doped capillary WGM laser^7^	20 mW, 980 nm laser	580 pm·min·mL^−1^		0–25 mL·min^−1^	Not available
This work	8 mW BBS 1500–1600 nm	342 pm·min·mL^−1^		0–5.04 mL·min^−1^	11.4 s

*CMOS* complementary metal-oxide-semiconductor, *MEMS* microelectromechanical systems, *PDMS* polydimethylsiloxane, *LED* light emitting diode, *BBS* broadband light source, *FBG* fiber Bragg grating, *WGM* whispering gallery mode.

It should be noted that our proposed sensor is composed from dielectric materials and as such offers the benefits of immunity to electromagnetic interference and is capable of remote operation in hard-to-reach, explosive, and chemically aggressive environments. Unlike the mechanical sensors, our proposed flowmeter has no moving parts and offers better stability against vibrations compared to some other technologies, e.g., a piezoelectric cantilever sensor, whose operation is easily disturbed by vibrations. For some fiber-based flowmeters, the complexity and high cost of fabrication, or their high potential for pollution of the sample, e.g., in the case of an FBG sensor head, have hindered their applications in microfluidics. In such cases, the unit of meters per second (m·s^−1^) was commonly utilized for characterizing the flowmeter performance, given the necessity of embedding the sensor within a flow tunnel of relatively large dimensions. In our case, a capillary with a small diameter was utilized for the airflow detection, demonstrating its suitability for application in air or gas detection characterized by small volumes of consumption. In addition, the definition of the sensitivity exhibits variability owing to the diverse fitting methods applied in certain cases involving flowrate sensors. The sensitivity of proposed sensor was determined by the slope of the fitting in the range from 0 to 2.52 sccm, whereas the sensitivity of the sensor described in [7] was derived by the differentiation of the fitting equation, and the value of 72 GHz·sccm^−1^ (~576 pm·sccm^−1^) at flowrate of 0.01 sccm was cited. Despite the response time being larger compared with some sensors listed in Table 1, the response time of the proposed sensor could be improved through the optimization of the structure parameters, such as the volume of LC involved in the heat transfer or the capillary wall thickness to increase the heat transfer efficiency. There have been few papers concerning the airflow detection based on WGM resonators in recent years. Our proposed scheme provides an advantage of using a low power laser, which contrasts with the sensors that either use a pulsed laser or a high-power continuous laser for excitation of thermal effects.

## Conclusions

A flowmeter based on a liquid crystal-filled nested capillary has been proposed and experimentally demonstrated. The WGMs were excited in the capillary by coupling light from the tapered fiber placed perpendicularly to the capillary. A nematic liquid crystal (MDA-05-2782) was studied as the thermo-optic sensitive filling material for the flowmeter. The spectral shift of the WGM resonances in the spectrum of the fiber taper was demonstrated when the air was passed through the inner capillary due to the temperature-induced changes of the RI of the LC caused by the cooling effect of the airflow. The electric field distribution within the coupled nested capillaries was first simulated, and the result showed that the changes in the field distribution within the structure do not influence the spectral shift of the WGM resonances due to changes in the surrounding RI. Then the simulation of the temperature distribution in the nested capillary demonstrated how the airflow in the inner capillary led to the temperature decrease of the LC. A flowmeter with a maximum sensitivity of 0.3423 nm·sccm^−1^ in the range of the flow rates from 0 sccm to 2.52 sccm and a resolution of 5.72 pm has been demonstrated in the experiment. The temperature change inside the nested capillary was investigated by analysing the temperature response of the proposed structure. As a result, the relationship between the flow rate and temperature changes in the nested capillary was estimated as 1.31 °C·sccm^−1^. To demonstrate the importance of the LC filling within the proposed structure, a similar experiment using an air-filled nested capillary was carried out, which resulted in negligible wavelength shift. By running the sensor at different conditions and temperatures, we observed that temperature increase deteriorates the sensing performance for our proposed flowmeter sensor. The nested capillary flowmeter also shows good stability, repeatability, and a competitive response time, facilitating its potential applications in real-time detection of a range of different gases and fluids. It should be noted that the dynamic range of the proposed sensor is limited by the diameter of the capillary since higher flow rates are difficult to achieve in thinner capillaries, especially for liquids with high viscosity. However, the proposed sensor shows potential for monitoring of gases and fluids in turbulent flow conditions, given the more effective heat transfer occurring in the case of turbulent flows compared with laminar flows. Some of the practical challenges that need to be overcome in practical applications of the proposed sensor include the need for maintaining constant temperature of the structure, ensuring better mechanical stability and development of a simple and low-cost interrogation.

## Data Availability

The data that support the findings of this study are available from the corresponding author upon reasonable request.
